# Precipitation and recovery of soy protein extracted in natural deep eutectic solvents: Emphasizing solvent reusability for sustainable processing

**DOI:** 10.1016/j.fochx.2026.104289

**Published:** 2026-08-06

**Authors:** Gulay Ozkan, Gulsah Karabulut

**Affiliations:** a Department of Food Engineering, Faculty of Chemical and Metallurgical Engineering, Istanbul Technical University, 34469, Maslak, Istanbul, Türkiye; b Department of Food Engineering, Faculty of Engineering, Sakarya University, 54187, Serdivan, Sakarya, Türkiye

**Keywords:** Choline chloride, Glycerol, Protein precipitation, Closed-loop, Structural properties, Solvent recycling, Antisolvent–isoelectric precipitation

## Abstract

Soy protein is one of the most important plant-based protein source used in food formulations due to its favorable nutritional profile and functional properties. However, the increasing interest in sustainable processing has led to the search for environmentally friendly extraction systems that can reduce reliance on conventional solvents. In this context, natural deep eutectic solvents (NADES) have attracted attention as effective media for plant protein extraction; however, protein recovery and solvent reuse after extraction still present significant practical challenges. In this study, soy proteins extracted from defatted soybean meal using choline chloride: glycerol NADES were recovered through isoelectric precipitation, antisolvent precipitation using water, ethanol, or water: ethanol (1:1) at extract: antisolvent ratios (1:2 to 1:6), and a sequential combined strategy involving pI adjustment followed by water: ethanol addition. Both the type of antisolvent and the dilution ratio had a marked influence on precipitation behavior. Water did not produce visible precipitation at low dilution ratios, whereas ethanol-containing systems more effectively facilitated protein aggregation. Among all approaches, the combined pI + water: ethanol (1:4) method gave the most favorable results, yielding a precipitation yield of 6.03%, a protein recovery of 77.47%, and the highest protein purity (88.60%). ATR–FTIR analysis showed that the main protein bands (Amide I–III) were preserved after recovery, while SEM images revealed irregular aggregated particles characteristic of proteins. In terms of solvent reuse, NADES recovery remained high over three consecutive cycles (96.4 % to 89.6 %), whereas extraction efficiency retention gradually declined. This reduction was accompanied by an increase in viscosity from 63.2 to 88.8 mPa·s, although no major structural changes were observed in the FTIR spectra of the recycled solvent. These findings indicate that combining charge neutralization with polarity modulation is an effective strategy for recovering high-purity soy protein and support the short-term reuse of NADES in a closed-loop extraction system.

## Highlights

• Soy protein recovery from natural deep eutectic solvents depended strongly on precipitation strategy.
• Combined pI and water–ethanol treatment gave the best overall performance.
• Recovered proteins retained their main Fourier transform infrared spectroscopy bands across all treatments.
• NADES was successfully reused for three consecutive recovery cycles.

## Graphical abstract

**Figure:**
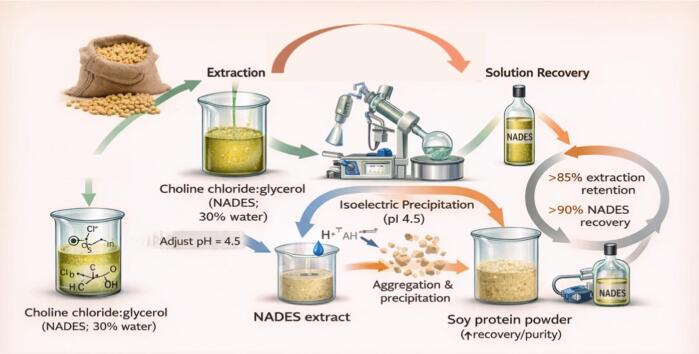

## 1 Introduction

In recent years, natural deep eutectic solvents (NADES) have attracted increasing attention as green extraction media for bioactive compounds, including proteins, owing to their negligible volatility, biodegradability, and tunable physicochemical properties, which facilitate cell-wall disruption and biomolecule solubilization (Huang et al., 2025). NADES are typically prepared by combining a hydrogen bond acceptor (HBA) with a hydrogen bond donor (HBD), generating a stable liquid phase through extensive hydrogen-bonding interactions (Hikmawanti et al., 2021). Among the available systems, choline chloride–based NADES, particularly choline chloride: glycerol mixtures, are appealing for food and pharmaceutical applications due to their low toxicity, biocompatibility, and broad regulatory acceptability of their constituents (Ahmed et al., 2026; Karabulut, Sanli and Ozkan, 2025).

In parallel, rising demand for sustainable, plant-based proteins has intensified interest in valorizing agricultural side streams and widely available crops such as soybean (*Glycine* max) (Upadhyay et al., 2026). Soy protein is valued for its nutritional profile, techno-functional performance, and versatility across foods, pharmaceuticals, and biomaterials (Landry & Ward, 2024). Conventional soy protein extraction commonly relies on alkaline solubilization followed by acid precipitation. While effective, these aqueous processes can promote protein denaturation, show limited selectivity, consume large volumes of water, and generate substantial effluents, factors that challenge alignment with green chemistry and circular bioeconomy objectives (Yadav et al., 2022).

Although NADES can efficiently solubilize plant proteins, practical implementation is constrained by downstream recovery and purification. Unlike dilute aqueous extracts, NADES matrices are often highly viscous and non-volatile, complicating precipitation, phase separation, and solvent removal (Raja Sekharan et al., 2021). In addition, the sustainability claim of NADES-assisted processing critically depends on solvent recycling; however, standardized, quantitative reuse protocols and performance benchmarks after recovery steps are still limited (Liang et al., 2023).

To date, protein recovery from deep eutectic solvents (DES)/NADES systems has mainly been approached using single-step strategies, including isoelectric precipitation (e.g., oat, mushroom, hemp), antisolvent precipitation with water and/or ethanol (cassava leaves, brewer's spent grain, sesame meal, kenaf seed, tiger nut meal), and dialysis-assisted solvent removal (zein, fava bean) (Anvar et al., 2025; Cao et al., 2023; Y. Chen et al., 2025; Ge et al., 2025; Karabulut et al., 2024; Patra and V, 2025). However, these strategies are most often evaluated independently, and studies commonly report extraction yield or recovery yield without a unified assessment of the purity and structural integrity of the recovered protein. Moreover, although DES/NADES physicochemical properties (e.g., pH, viscosity, conductivity, Fourier transform infrared spectroscopy signatures) are frequently documented, systematic comparisons of combined recovery routes, such as controlled antisolvent addition coupled with isoelectric precipitation, and their consequences on yield–purity trade-offs and protein structure remain scarce. Importantly, solvent reusability after dilution-intensive precipitation steps is rarely quantified, leaving a critical gap for designing closed-loop, environmentally credible NADES-based protein extraction processes (Didion et al., 2025; Duong-Ly & Gabelli, 2014; Liang et al., 2023).

In this context, the present study develops and analyzes a precipitation and recovery strategy for soy protein extracted in choline chloride: glycerol NADES, with particular emphasis on solvent recovery and multi-cycle reuse. Single and combined precipitation routes were systematically investigated, including: (i) isoelectric precipitation, (ii) antisolvent precipitation using water, ethanol, and water: ethanol mixtures at different ratios, and (iii) their sequential combinations. Recovered proteins were characterized in terms of yield, recovery, purity, and chemical structure, while the post-precipitation NADES was evaluated for recovery efficiency and extraction performance retention over repeated cycles. By integrating downstream selectivity with solvent reuse metrics, this work addresses key technical and sustainability requirements for scalable NADES-assisted plant protein biorefineries.

## 2 Materials and methods

### 2.1 Materials

Soybean meals (*Glycine* *max*) (47.13 % protein; 1.22 % oil on a dry basis) were obtained from Sunar Mısır in Adana, Türkiye. The meals were ground using a laboratory mill (mesh size 0.5 mm). The soy meals were dried in an oven at 40 °C for 24 h to remove residual solvent and stored in airtight containers at 4 °C until further use.

Choline chloride (≥98 %), glycerol (≥99.5 %), ethanol (absolute, ≥99.8 %), sodium chloride (NaCl), ammonium sulfate ((NH₄)₂SO₄), hydrochloric acid (HCl), and sodium hydroxide (NaOH) were of analytical grade and were purchased from Sigma-Aldrich (St. Louis, MO, USA). Ultrapure water (18.2 MΩ·cm) was used throughout all experiments.

### 2.2 Preparation of natural deep eutectic solvent (NADES)

The NADES was prepared by mixing choline chloride and glycerol (ChCl: Gly) at a 1:2 mole ratio, since this composition exhibited the highest extraction efficiency among the NADES formulations previously evaluated (Karabulut, Sanli, et al., 2025). Specifically, 13.96 g of choline chloride (pre-dried at 50 °C during 12 h) and 18.42 g of glycerol were weighed and combined in a 100 mL glass beaker. The mixture was stirred magnetically at 80 °C for approximately 30 min until a homogenous, transparent, and slightly viscous liquid was obtained. After cooling to room temperature, distilled water was added at 30% (*v*/v) of the total NADES volume to reduce viscosity and improve mass transfer during extraction. The hydrated NADES was gently stirred until uniform and used as the working solvent. To minimize moisture variability, the preparation vessel was covered with parafilm during cooling. The final NADES was stored in sealed amber glass bottles at room temperature until use.

#### 2.2.1 Characterization of NADES

The physicochemical properties of the prepared NADES were characterized prior to extraction experiments. pH was measured at 25 °C using a calibrated digital pH meter (Seven Compact S210; Mettler-Toledo, Switzerland). Viscosity (mPa·s) was measured at 25 °C using a rotational rheometer (Fungilab Alpha L Smart Series, Spain) equipped with appropriate spindle geometry under controlled shear rate conditions. Refractive index was recorded using an Abbe refractometer at 25 °C. FTIR spectroscopy (Perkin Elmer Spectrum Two, USA) (4000–400 cm^−1^) was performed to confirm hydrogen bond formation between choline chloride and glycerol. The broadening and shifting of O—H stretching bands (3200–3500 cm^−1^) and changes in C—N and C—O vibration regions indicated the successful formation of the eutectic system. All measurements were performed in triplicate.

### 2.3 Extraction of soy protein using NADES

For the extraction of soy proteins, 5.0 g of defatted soy flour was suspended in 50 mL of pre-heated ChCl: Gly based NADES (10% *w*/*v* solid-to-solvent ratio). The extraction was carried out at 50 °C for 4 h under continuous magnetic stirring at 150 rpm using a temperature-controlled water bath (Memmert SV1422; Memmert GmbH & Co. Nürnberg, Germany). Following extraction, the slurry was cooled to room temperature and centrifuged at 13000 x*g* for 20 min (25 °C) (Megafuge 8R; Thermo Scientific, Darmstadt, Germany). The supernatant, referred to as NADES protein extract, was carefully collected for subsequent precipitation studies. The pellet (residual solids) was discarded (Karabulut, Sanli, et al., 2025).

### 2.4 Protein precipitation and recovery

Multiple strategies, including single and combined precipitation techniques, were investigated to recover soy protein from the ChCl: Gly-based extract effectively. Isoelectric precipitation was performed at room temperature because protein aggregation is primarily triggered by charge neutralization at the isoelectric point. To further reduce protein solubility and increase precipitation efficiency while minimizing potential protein denaturation, antisolvent-based experiments were conducted at 4 °C.

#### 2.4.1 Isoelectric precipitation (pI method)

The pH of the ChCl: Gly based protein extract was adjusted to the isoelectric point (pI) of soy proteins, which is around pH 4.5, using 1 M HCl added dropwise under gentle stirring. The pH was monitored continuously using a calibrated pH meter (pH Meter Bench Type pH/mV/Temp HI-2211 Made in Hanna Romania). Once the target pH was reached, the sample was incubated at room temperature (22–25 °C) for 1 h to allow protein aggregation and sedimentation. The resulting precipitate was separated by centrifugation at 13000 x*g* for 15 min at 4 °C, and washed three times with distilled water. After, the collected precipitate was redispersed in distilled water, neutralized to pH 7.0 using 2 N NaOH, and subjected to freeze-drying for 48 h (ALPHA 1–2 LD plus; Martin Christ Gefriertrocknungsanlagen GmbH, Osterode am Harz, Germany) (Verfaillie et al., 2023). Soluble protein content of the precipitate was determined using the Bradford assay as explained in section 2.5.1 (Bradford, 1976).

#### 2.4.2 Antisolvent precipitation

Three antisolvent systems were evaluated for their ability to reduce protein solubility and promote precipitation: (i) Ultrapure water (W; 100%), (ii) Absolute ethanol (E; 100%), and (iii) Water: ethanol mixture (WE; 1:1, *v*/v). The selected antisolvent was added dropwise to the ChCl: Gly-based protein extract under gentle stirring at volume ratios of 1:2, 1:4, and 1:6 (v/v, ChCl: Gly-based extract: antisolvent). After complete addition, the mixtures were allowed to stand at 4 °C for 1 h to ensure full precipitation. Precipitates were recovered by centrifugation (13,000 x*g*, 15 min, 4 °C) and washed three times with distilled water. After, the collected precipitate was redispersed in distilled water, neutralized to pH 7.0 using 2 N NaOH, and subjected to freeze-drying for 48 h (ALPHA 1–2 LD plus; Martin Christ Gefriertrocknungsanlagen GmbH, Osterode am Harz, Germany) (Patra & Prasath, 2025; Karabulut et al., 2024; Hadinoto et al., 2025; Ge et al., 2024). The soluble protein content of the precipitates was determined using the Bradford assay as explained in section 2.5.1 (Bradford, 1976). The preferred antisolvent type and its ratio were selected according to the results of precipitation yield and recovery rate.

#### 2.4.3 Combined isoelectric and antisolvent precipitation

To exploit the synergistic effects of pI and solvent polarity change, the pH of the ChCl: Gly based extract was first adjusted to 4.5 using 1 M HCl, as described in Section 2.4.1. The acidified extract was kept at 4 °C for 1 h to promote initial protein aggregation. Without removing the formed aggregates, antisolvent was then added at a fixed NADES extract: antisolvent ratio (*v*/v) using the selected antisolvent system (WE, 1:1 v/v; extract:antisolvent = 1:4). The mixture was stirred for 10 min and kept at 4 °C for 1 h. The resulting precipitate was separated by centrifugation at 13000 x*g* for 15 min at 4 °C, and washed three times with distilled water. After, the collected precipitate was redispersed in distilled water, neutralized to pH 7.0 using 2 N NaOH, and subjected to freeze-drying for 48 h (ALPHA 1–2 LD plus; Martin Christ Gefriertrocknungsanlagen GmbH, Osterode am Harz, Germany). Soluble protein content of the precipitates was determined using the Bradford assay as explained in section 2.5.1 (Bradford, 1976).

### 2.5 Analytical methods

#### 2.5.1 Protein content determination

Protein concentration in NADES extracts and recovered protein fractions was first determined using the Bradford colorimetric assay (Bradford, 1976) to quantify soluble protein. A calibration curve was prepared using bovine serum albumin (BSA) as the standard within the range of 0–1.0 mg mL^−1^ (R^2^ > 0.99). Briefly, 150 μL of the supernatant was mixed with 3 mL of Bradford reagent, incubated for 10 min at room temperature, and the absorbance was measured at 595 nm using a UV–Vis spectrophotometer (Shimadzu UV-1240, Kyoto, Japan). To account for potential interference from NADES components, a blank sample of the same choline chloride composition without protein was analyzed in parallel, and the absorbance value of this sample was subtracted from the sample measurements before determining the protein quantity.

Protein recovery was calculated to evaluate the efficiency of protein transfer from the NADES extract to the precipitated protein fraction (Eq. 1):

(1) $$\text{Protein recovery}\;\left(\%\right)=\frac{\text{Protein content in recovered fraction}}{\text{Protein content in NADES extract}}\times 100$$

Precipitation yield, in contrast, was calculated based on mass balance to assess the overall yield of solid protein obtained after precipitation. Yield was expressed as the ratio of the mass of recovered protein to the total extractable protein content (Eq. 2):

(2) $$\text{Yield}\;\left(\%\right)=\frac{\text{Mass of recovered protein}\;}{\text{Total extractable protein}\;}\times 100$$

#### 2.5.2 Protein purity determination

Protein purity of the recovered soy protein fractions was determined by measuring total nitrogen content using the Dumas combustion method (Karabulut, Unlu, et al., 2025). Approximately 100–150 mg of freeze-dried protein sample was accurately weighed and analyzed using a Dumas nitrogen analyzer (Velp NDA 701, Italy). The sample was combusted at high temperature (900–1000 °C) in the presence of oxygen, converting nitrogen-containing compounds into nitrogen gas (N₂), which was quantified by a thermal conductivity detector. The nitrogen content (%) obtained from the analysis was converted to crude protein content using a nitrogen-to-protein conversion factor of 6.25. All measurements were performed in triplicate.

#### 2.5.3 Fourier transform infrared (FTIR) spectroscopy

The FTIR spectrum of the protein and NADES was recorded using an Attenuated Total Reflection (ATR)-FTIR spectrometer (PerkinElmer Spectrum Two, USA) equipped with a diamond crystal, operating under ambient temperature and pressure conditions. FTIR spectral data were recorded in the range of 4000–600 cm^−1^ with a resolution of 4 cm^−1^, averaging 16 scans per sample to improve signal-to-noise ratio. The spectra were analyzed to identify characteristic functional group vibrations, with particular attention to the Amide I (∼1650 cm^−1^), Amide II (∼1540 cm^−1^), and Amide III (∼1235 cm^−1^) bands.

#### 2.5.4 Morphological observation

The morphological properties of proteins were examined using scanning electron microscopy (SEM; S-4700, Hitachi, Japan) operated at 15 kV. Proteins were mounted on double-sided conductive carbon adhesive tape, cut to expose the internal structure, and sputter-coated with a gold–palladium (Au—Pd) layer prior to imaging. Micrographs were acquired at ×500 magnification.

#### 2.5.5 NADES recovery and reusability

After protein precipitation using the selected combined pI and antisolvent approach (WE, 1:1 *v*/v), the residual NADES phase (soy meal to precipitation solvent ratio of 1:4, *w*/*v*) was separated by centrifugation at 13000 ×*g* for 15 min at 4 °C. The resulting supernatant, containing the NADES, was carefully collected and subjected to solvent recovery. The recovered NADES was readjusted to its initial pH before reuse to minimize carryover effects from the acidification step. Residual water and ethanol were removed by rotary evaporation (Büchi R-100, Germany) at 40 °C under reduced pressure until the majority of volatile components was eliminated. The concentrated NADES was subsequently dried under mild vacuum at 50 °C for 2–3 h to obtain a clear and viscous NADES residue suitable for further characterization and reuse. The mass of the recovered NADES was recorded, and recovery efficiency (%) was calculated as (Eq. 3):

(3) $$\text{NADES recovery efficiency}\;\left(\%\right)=\frac{\text{Mass of recovered NADES after drying}\;}{\text{Initial mass of NADES before extraction}}\times 100$$

No additional choline chloride or glycerol was supplemented during the reuse cycles in order to evaluate intrinsic solvent stability.

For reusability evaluation, the recovered and dried NADES was gently reheated to 80 °C under continuous magnetic stirring for 10–15 min until a homogeneous liquid was obtained. The reconstituted NADES was then cooled to 50 °C and directly reused for a subsequent protein extraction cycle under the same conditions described in Section 2.3. To quantify the performance of the recycled solvent, extraction efficiency retention (%) was calculated as the ratio of protein extracted in reused NADES to that extracted in fresh NADES under identical conditions (Eq. 4).

(4) $$\text{Extraction efficiency retention}\;\left(\%\right)=\frac{\text{Protein extracted in reused NADES}\;}{\text{Protein extracted in fresh NADES}}\times 100$$

NADES reusability was assessed over three consecutive extraction–precipitation cycles. After each cycle, solvent recovery and drying were performed as described above. The performance of the recycled NADES was evaluated by monitoring protein yield and solvent properties, including pH, viscosity, refractive index (RI), and FTIR spectra to assess potential structural changes during repeated use. In addition, FTIR spectra were recorded after each cycle to assess potential chemical changes and to verify structural integrity of the NADES during repeated use.

### 2.6 Statistical analysis

The data was analyzed and shown using IBM SPSS Statistics 20 and Origin 19 software, respectively. One-way analysis of variance (ANOVA) was employed, and the Tukey test was selected to evaluate the significance of differences (*p <* 0.05). The results are presented as mean ± standard deviation (SD).

## 3 Results and discussion

### 3.1 Precipitation performance of soy protein from ChCl: Gly based extract

**Fig. 1:**
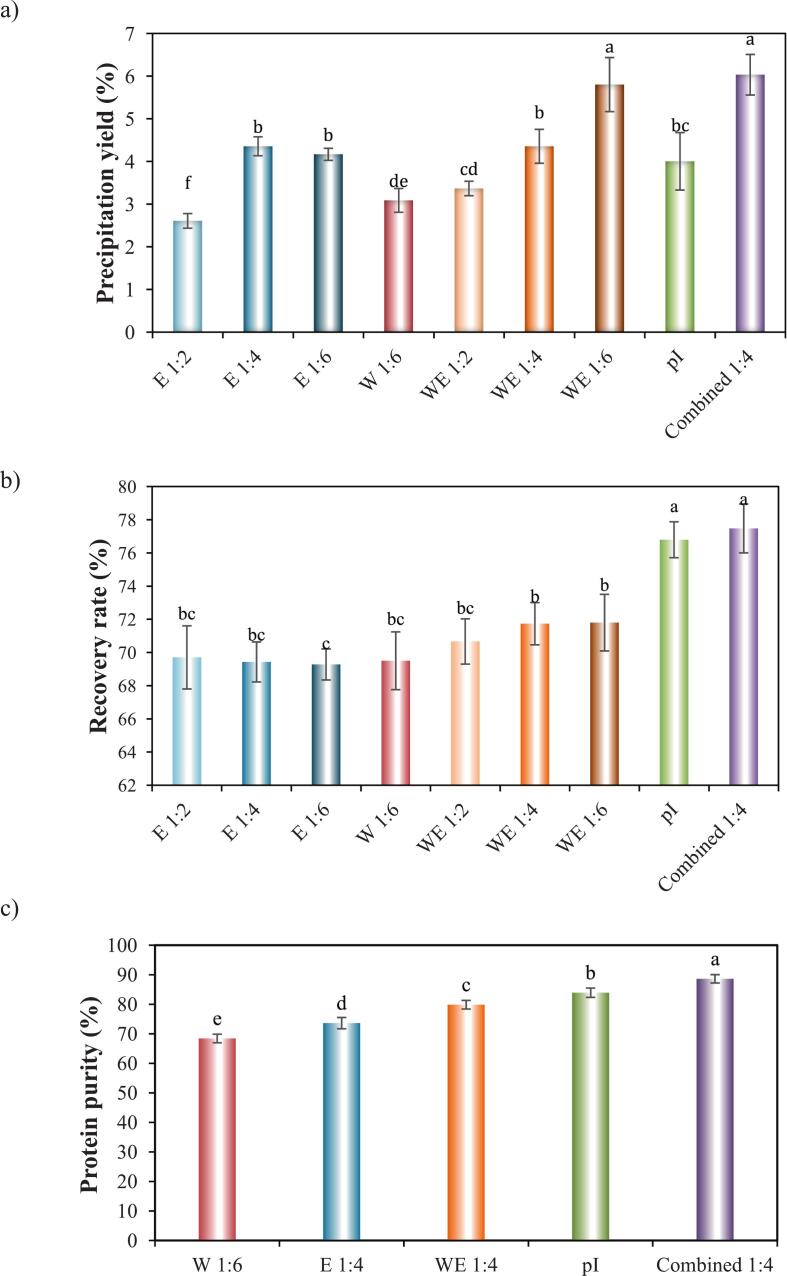
Precipitation yield (%) (a), protein recovery rate (%) (b), and protein purity (%) (c) of soy protein precipitates obtained from choline chloride: glycerol NADES extracts using different precipitation strategies (W, E, WE, pI, and pI+WE). Sample codes: W (water antisolvent), E (ethanol antisolvent), WE (water–ethanol mixture), pI (isoelectric precipitation at pH 4.5), and Combined (sequential isoelectric precipitation followed by antisolvent addition).

In the current study, precipitation of soy protein from a choline chloride:glycerol (1:2)-based NADES extract was systematically evaluated by comparing different precipitation strategies and extract:antisolvent ratios. The effects of precipitation type and dilution ratio on precipitation yield and recovery rate are indicated in Fig. 1a-1b.

Among the individually tested precipitation approaches, the WE mixture at a 1:6 extract:antisolvent ratio resulted in the highest precipitation yield (5.801 ± 0.634 %, *p* < 0.05), followed by WE 1:4 > E 1:4 > E 1:6 > pI > WE 1:2 > W 1:6 > E 1:2. Apart from those, precipitation recovery rates ranged between 69.28 and 76.79%, with isoelectric precipitation yielding the highest recovery (76.79 ± 1.08 %, *p* < 0.05). However, despite its relatively high recovery efficiency, isoelectric precipitation alone did not produce the highest precipitation yield, suggesting incomplete disruption of solvent–protein interactions within the NADES matrix.

Notably, when water alone was used as an antisolvent at lower dilution ratios (1:2 and 1:4), no visible protein precipitation was recorded. This behavior can be attributed to the strong hydrogen-bonding network between choline chloride and glycerol, which maintains protein solubilization under moderate dilution. In contrast, a precipitate was only obtained at the highest dilution tested (W 1:6), indicating that extensive dilution is required for water to sufficiently disrupt the NADES solvation environment and trigger phase separation. Ethanol-containing antisolvent systems are expected to facilitate precipitation more readily by lowering the dielectric constant and weakening solvent–protein interactions, thereby promoting aggregation and sedimentation.

Based on the overall performance, the WE mixture at a 1:4 ratio was selected for combination with isoelectric precipitation, as it represented the most practical operating point with a favorable balance between precipitation yield and recovery efficiency, despite the absence of statistically significant differences in recovery rate among the tested WE ratios. The sequential application of charge neutralization (pH adjustment to pI) followed by dielectric constant reduction (WE addition) led to a significant enhancement in precipitation performance. Under combined conditions, precipitation yield increased to 6.032 ± 0.475 %, while recovery rate improved to 77.47 ± 1.47 % (*p* < 0.05). No statistically significant differences in recovery rate were observed among the tested extract-to-water–ethanol (WE) antisolvent ratios (1:2–1:6), suggesting a plateau in recovery efficiency within this range. It should be noted that precipitation yield and recovery rate represent different evaluation bases, with the former calculated relative to the initial raw material and the latter based on the protein content in the NADES extract. These findings indicate a combined/sequential approach between polarity modulation and electrostatic destabilization mechanisms.

Deep eutectic solvents have gained considerable attention as green extraction media due to their high solubilization capacity and potential recyclability, as previously reported in the literature (Jia et al., 2020). However, downstream protein recovery remains a major bottleneck. Protein recovery from DES-based extracts is commonly achieved by different precipitation approaches, including antisolvent-induced precipitation, isoelectric precipitation, and salt-induced precipitation. Antisolvent precipitation operates primarily through disruption of HBA–HBD hydrogen bonding and alteration of solvent polarity by addition of solvents such as ethanol, acetone, or water, whereas isoelectric precipitation relies on minimizing net protein charge. Salt-induced precipitation, such as ammonium sulfate addition, promotes protein aggregation via salting-out effects. Following precipitation, the antisolvent can be removed by evaporation or distillation, allowing the DES to be recovered and reused (Hanafi et al., 2024).

Despite numerous studies investigating the effects of many parameters, including extraction time, temperature, DES type, HBA: HBD molar ratio on the extraction efficiency of the target compounds (Hansen et al., 2021), there is no comprehensive understanding about the effectiveness of precipitation types for the protein recovery. Instead, in the previous studies, protein has generally been obtained using a single precipitation method. For instance, Karabulut, Sanli, et al. (2025) recovered sunflower proteins from a choline chloride:glycerol (1:2) NADES using distilled water (1:5), reporting a relatively low recovery (∼15 %). Similarly, (Grudniewska & Pastyrczyk, 2023) investigated protein recovery from spent hops using different choline chloride–based DES systems (1:2) and applied distilled water as an antisolvent at 3–5-fold volumes of the extract. The highest protein yields were obtained using choline chloride:lactic acid (2.98 g/100 g meal) and choline chloride:ethylene glycol (2.83 g/100 g meal), whereas lower yields were observed for choline chloride:propylene glycol (2.20 g/100 g meal) and choline chloride:glycerol (2.11 g/100 g meal). These results indicate that both DES composition and the applied precipitation strategy strongly influence protein recovery efficiency. In another study, (Zheng et al., 2025) also employed water as an antisolvent for potato protein recovery; however, precipitation conditions were not comprehensively described, limiting mechanistic interpretation.

Alternative precipitation methods have shown moderate improvements employed ammonium sulfate precipitation following dilution of NADES-based extracts (1:10, *v*/v) for protein recovery from *Moringa oleifera* seeds, achieving extraction efficiencies of approximately 30% for choline chloride-malic acid and choline chloride-urea systems (Wang et al., 2026). Likewise, Karabulut et al. (2024) used ethanol as an antisolvent at four times the extract volume to precipitate proteins from mushroom stems extracted with choline chloride:glycerol (1:2) and choline chloride:lactic acid (1:2), reporting recovery rates of 22.04 % and 21.47 %, respectively. Although these studies confirm the feasibility of antisolvent-based precipitation, recovery efficiencies remain considerably lower than those obtained in the present work. Hadinoto et al. (2025) reported a high extraction yield (42 %) for proteins recovered from brewer's spent grain using a choline chloride–trehalose NADES combined with isoelectric precipitation, highlighting the effectiveness of pH-induced precipitation for protein recovery. In comparison, conventional alkaline extraction of soybean proteins from different soybean varieties has been reported to yield 58.7–66.9 %, while acid precipitation yields 50.8–57.8 % (Verfaillie et al., 2023), indicating that the recovery efficiency achieved in the present NADES-based combined strategy (>77 %) is comparable to or higher than conventional aqueous extraction systems.

Compared to the aforementioned studies, the present work demonstrates that a combined precipitation strategy significantly enhances protein recovery from choline chloride:glycerol (1:2) NADES extracts. The observed recovery rate exceeding 77 % represents a substantial improvement over most previously reported values. These results clearly indicate that precipitation strategy, rather than extraction efficiency alone, is the critical determinant of overall protein recovery from ChCl:Gly based systems. The integration of dielectric modulation and electrostatic destabilization provides a rational and efficient framework for maximizing protein recovery while maintaining compatibility with solvent recycling and sustainable processing objectives.

### 3.2 Protein purity of NADES precipitation

Protein purity differed significantly among precipitation strategies (*p* < 0.05), as shown in Fig. 1c. The combined approach (pI + WE (1:4)) yielded the highest purity (88.6 ± 1.41%), followed by pI (83.9 ± 1.55 %), WE 1:4 (79.8 ± 1.45 %), E 1:4 (73.6 ± 1.94 %), and W 1:6 (68.4 ± 1.45 %). The purity achieved in the combined system can be mechanistically attributed to the sequential destabilization of protein–solvent interactions through dual pathways (Mattice & Marangoni, 2020). The initial addition of the WE mixture likely reduced the effective dielectric constant of the medium while simultaneously weakening the hydrogen-bonding network between choline chloride and glycerol (Hanafi et al., 2024). This step may have partially disrupted the NADES-mediated solvation environment surrounding soy proteins and promoted controlled desolvation before pI adjustment. Subsequent adjustment to the isoelectric point would further minimize electrostatic repulsion among protein molecules, thereby enhancing selective aggregation of charge-neutralized protein fractions (Verfaillie et al., 2023; Yao et al., 2023). Thus, the integration of polarity modulation and charge neutralization appears to improve protein precipitation selectivity while limiting the co-precipitation of low-molecular-weight solutes and residual solvent components (Belesov et al., 2025; Q. Chen et al., 2021).

In contrast, when water alone was used as an antisolvent (W), the lowest purity was obtained. Although dilution weakens the NADES structure, the highly polar environment created by water may favor the co-precipitation or entrapment of hydrophilic non-protein compounds and partially solvated NADES constituents, thereby reducing selectivity (Jiang et al., 2024). Ethanol-based systems improved purity compared to water, likely because the reduced solvent polarity lowered protein solubility and promoted aggregation through desolvation; however, ethanol-induced aggregation may be less selective than isoelectric precipitation and can also entrap co-extracted biomolecules within the precipitated protein matrix (Cao et al., 2023). This interpretation is also consistent with reports that aqueous ethanol-based fractionation can alter soy protein composition and enrich protein fractions while still allowing the retention of some non-protein material, depending on the separation pathway, similarly reported by (Peng et al., 2021).

pI alone demonstrated relatively high purity, confirming that charge neutralization remains a dominant driver of selective protein aggregation (Yao et al., 2023). Nevertheless, the slightly lower purity compared to the combined approach suggests that, in a highly structured NADES matrix, pH adjustment alone may not fully disrupt protein–NADES hydrogen-bond interactions. Therefore, polarity modification prior to pI adjustment appears to facilitate more efficient release of solvated proteins from the eutectic environment. This interpretation is in agreement with studies of (Verfaillie et al., 2023) and (Q. Chen et al., 2021), showing that the isoelectric precipitation step can strongly affect the composition and physical properties of soy protein isolates and that recovery conditions in DES-based soy protein extraction need to be specifically tailored for efficient and selective precipitation. Importantly, the observed purity trend also reflects the intrinsic trade-off between yield and selectivity. Strategies relying mainly on antisolvent dilution may enhance precipitation yield but compromise purity due to co-aggregation phenomena. Conversely, the combined approach achieved both high recovery and elevated purity, indicating a balanced optimization of thermodynamic destabilization and electrostatic control. More broadly, this trend supports the view that DES/NADES-based protein recovery is governed by the interplay between solvent environment, precipitation route, and downstream selectivity rather than by a single destabilization mechanism alone.

### 3.3 FTIR spectroscopy of proteins recovered by different precipitation strategies

**Fig. 2:**
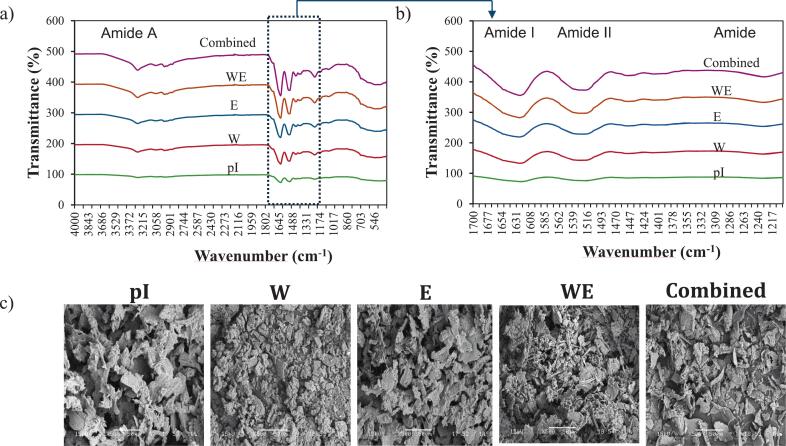
FTIR spectra (a, b) and SEM microstructures (c) of soy protein precipitates obtained from choline chloride:glycerol NADES extracts using different precipitation strategies (W, E, WE, pI, and pI+WE). Sample codes: W (water antisolvent), E (ethanol antisolvent), WE (water–ethanol mixture), pI (isoelectric precipitation at pH 4.5), and Combined (sequential isoelectric precipitation followed by antisolvent addition).

The FTIR spectra of the soy protein powders recovered through different precipitation routes were highly comparable, indicating that the main functional groups of the proteins were largely retained after precipitation (Fig. 2a–b). In protein-based systems, FTIR primarily reflects the local environment of functional groups and hydrogen-bonding patterns; therefore, it should be considered supportive rather than conclusive evidence of precipitation selectivity or structural rearrangement (Barth, 2007).

All samples showed a broad absorption band in the ∼3200–3400 cm^−1^ region (Amide A), mainly associated with N—H stretching overlapped with O—H stretching from hydrogen-bonded water. Weak bands were also observed in the ∼2920–2850 cm^−1^ region, corresponding to aliphatic C—H stretching of –CH₂/–CH₃ groups. The spectra were dominated by the characteristic amide bands of proteins, including Amide I (∼1650–1630 cm^−1^), arising mainly from C=O stretching of peptide bonds, Amide II (∼1545–1530 cm^−1^), related to N—H bending coupled with C—N stretching, and Amide III (∼1240–1300 cm^−1^), further confirming the presence of the protein backbone (Barth, 2007). Bands in the ∼1450–1400 cm^−1^ region may be assigned to CH bending and/or symmetric stretching of carboxylate groups, while the broad ∼1000–1150 cm^−1^ region may include contributions from C—O and C–O–C stretching associated with residual carbohydrate-like compounds or other co-extracted non-protein components (X. Chen et al., 2013).

Although the main bands were present in all samples, some differences in band breadth and relative intensity were observed among precipitation routes. These variations may reflect differences in hydrogen bonding, hydration, and protein–solvent interactions rather than major chemical changes in the protein backbone. For example, water-precipitated samples exhibited a relatively broader band in the ∼3200–3400 cm^−1^ region, which may be associated with a greater contribution of bound water and a stronger hydrogen-bonding environment within the dried aggregates. In contrast, samples recovered using ethanol-containing antisolvent systems (E and WE) showed slightly sharper amide regions, which may be related to altered solvation conditions during precipitation and subsequent aggregate formation. Similar effects have been reported in ethanol-based soy protein processing systems (Peng et al., 2021).

The spectra of the pI and combined treatments also remained broadly similar to those of the other samples, with no clear appearance of new bands or disappearance of characteristic protein bands. Slight differences in the Amide I–II region and in the ∼1000–1150 cm^−1^ region were observed, but these should be interpreted cautiously. Such changes may indicate differences in the local molecular environment and in the relative contribution of co-precipitated non-protein components, which is broadly consistent with the purity differences observed among precipitation routes. However, FTIR alone does not provide direct evidence for selective protein aggregation or compositional fractionation. Overall, the FTIR results suggest that the precipitation strategy did not cause major chemical alterations in the recovered soy protein powders, while modest differences in hydrogen bonding, hydration, and sample composition may still have occurred depending on the recovery route.

### 3.4 Morphological analysis proteins recovered by different precipitation strategies

SEM micrographs in Fig. 2c showed that all recovered soy protein powders exhibited irregular, fragmented, and aggregated structures, which are commonly observed in dried plant protein isolates. Overall, the micrographs confirmed that the recovered materials had heterogeneous particulate morphologies, although some visual differences in compactness and surface texture were noticeable among precipitation routes.

Water-precipitated samples appeared relatively more open and loosely packed, with visible voids between aggregates. In contrast, samples recovered using ethanol tended to exhibit somewhat denser and more clustered morphologies. The water–ethanol mixture produced structures with an intermediate appearance. The pI-precipitated sample also appeared slightly more cohesive, while the combined treatment showed a morphology broadly similar to that of the pI route. These visual differences may be associated with the distinct precipitation environments, which can influence aggregate formation, packing behavior, and the final structure of dried powders. SEM provides morphological information only and does not directly reveal molecular-level structural changes or precipitation selectivity. Therefore, the SEM results are better considered as qualitative support for route-dependent differences in aggregate organization rather than as direct evidence of changes in protein structure. In general, the images suggest that the precipitation strategy influenced the physical arrangement of the recovered particles to some extent, but all samples remained within the typical morphological range reported for precipitated plant protein powders (Hewage et al., 2024; Karimi et al., 2024).

### 3.5 NADES reusability and extraction performance

**Fig. 3:**
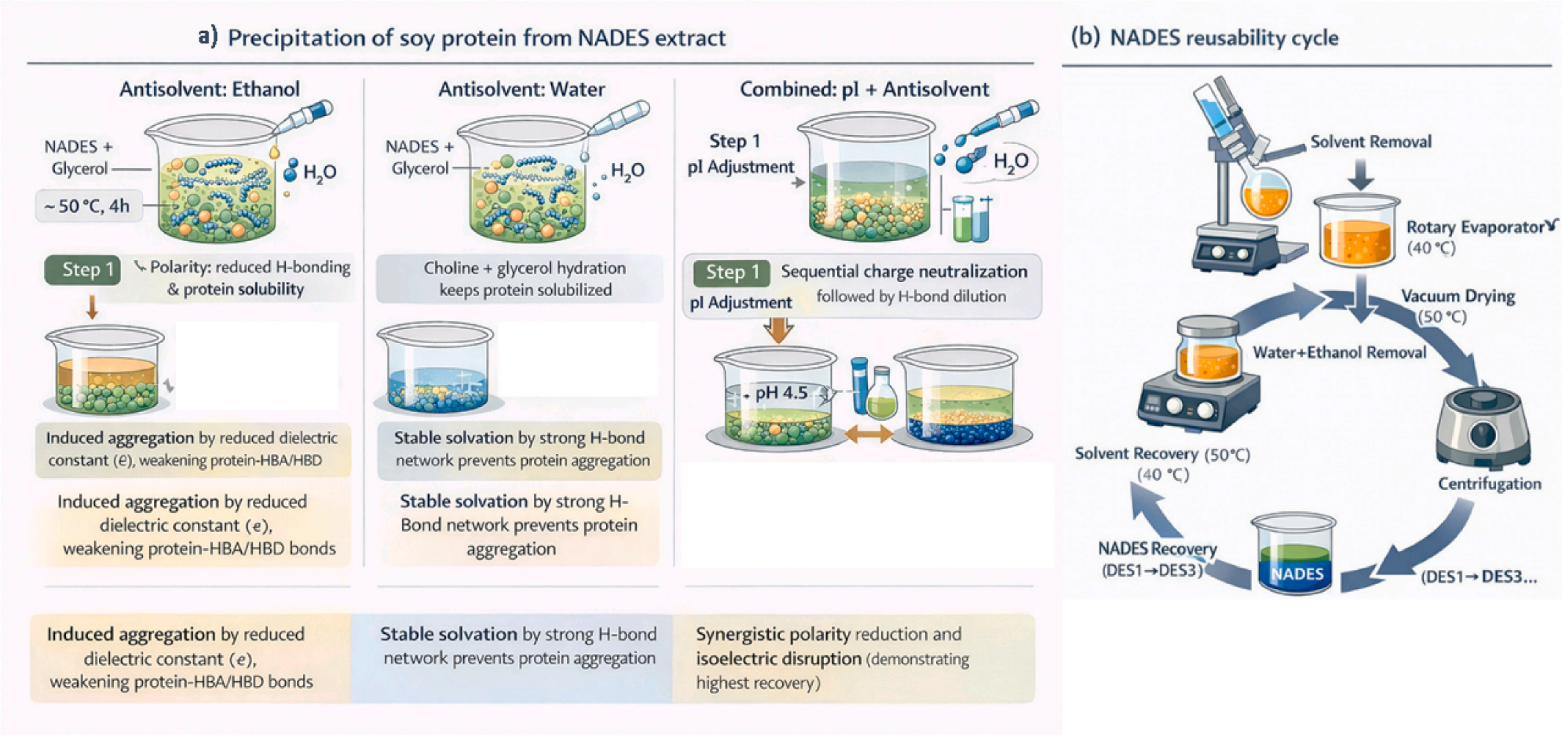
Protein precipitation from natural deep eutectic solvent (NADES) extracts (a) and NADES recycling process (b). The NADES system consisted of choline chloride:glycerol (ChCl: Gly), where choline chloride served as the hydrogen bond acceptor (HBA) and glycerol served as the hydrogen bond donor (HBD). Ethanol, water, and combined pI–antisolvent treatments were evaluated for protein recovery, followed by solvent recovery and reuse.

To support a closed-loop NADES-based protein biorefinery concept, solvent recovery and reuse were evaluated after the selected precipitation route. The process scheme (Fig. 3b) summarizes the extraction–precipitation workflow and the downstream solvent-recovery steps (rotary evaporation followed by vacuum drying) prior to reuse.

**Fig. 4:**
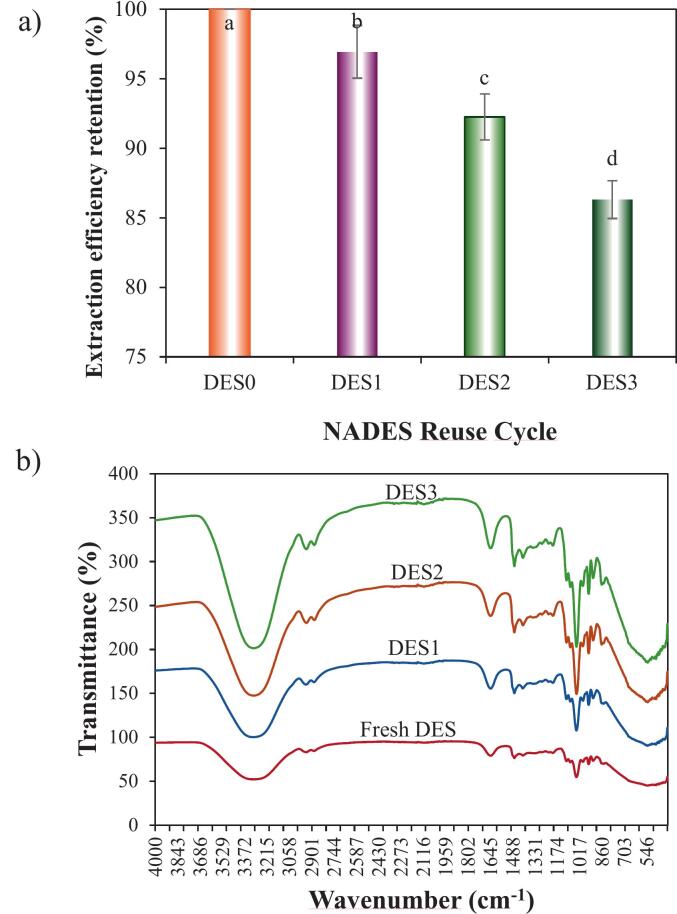
Extraction efficiency retention (%) (a) and FTIR spectra (b) in recovered NADES media. Fresh NADES was coded as DES0, whereas the recycled NADES samples obtained after the first, second, and third cycles were designated as DES1, DES2, and DES3, respectively. Different letters indicate significant differences, *p* < 0.05.

As shown in Fig. 4a, the extraction capacity of the recycled NADES decreased progressively with consecutive cycles (*p* < 0.05). Using fresh NADES (DES0) as the 100% reference, extraction efficiency retention was 96.8 % (1.cycle-DES1), 92.2 % (2.cycle-DES2), and 86.3 % (3.cycle-DES3), indicating a measurable but gradual loss of performance over three reuse cycles. This decline occurred despite the relatively high mass-based solvent recovery values reported in Table 1, which decreased from 96.4% (DES1) to 89.6 % (DES3). Together, these results suggest that the recycled solvent remained largely recoverable, but its functional extraction performance was partially compromised during reuse. A comparable trend has also been reported in a recent NADES-based protein extraction study on *Tenebrio molitor*, where reused NADES maintained substantial functionality over the initial cycles, but protein hydrolysis gradually decreased from 61 % in the first cycle to 58 % and 48 % in the second and third cycles, respectively, indicating that solvent reusability may be accompanied by progressive performance loss upon repeated use (Muñoz-Seijas et al., 2025).

**Table 1:** Recovery efficiency (%) and physicochemical properties (pH, refractive index, and viscosity) of choline chloride:glycerol NADES after three consecutive reuse cycles.⁎⁎

NADES cycle	Sample code	Recovery efficiency (%)	pH	Refractive index	Viscosity
1st cycle of NADES	DES1	96.4 ± 0.86^a^	6.08 ± 0.04^a^	1.4166 ± 0.0009^c^	63.2 ± 3.4^c^
2nd cycle of NADES	DES2	93.5 ± 0.90^b^	6.01 ± 0.04^a^	1.4186 ± 0.0009^b^	75.1 ± 3.6^b^
3rd cycle of NADES	DES3	89.6 ± 1.15^c^	5.92 ± 0.03^b^	1.4218 ± 0.0010^a^	88.8 ± 4.7^a^

⁎⁎ Mean ± SD; different letters indicate significant differences, *p* < 0.05.

The physicochemical properties of the recovered NADES provide mechanistic insight into this trend (Table 1). The most pronounced change was a steady increase in viscosity from 63.2 ± 3.4 mPa·s (DES1) to 88.8 ± 4.7 mPa·s (DES3), which is expected to hinder diffusion and solid–liquid mass transfer, thereby limiting protein solubilization during subsequent extractions. In parallel, the refractive index increased from 1.4166 ± 0.0009 to 1.4218 ± 0.0010, while pH decreased slightly from 6.08 ± 0.04 to 5.92 ± 0.03, pointing to modest compositional shifts in the recycled solvent, potentially associated with gradual moisture changes and accumulation of non-precipitated solutes. Similar explanations have been proposed in the literature, where repeated NADES recycling was associated with declining extraction or hydrolysis efficiency due to incomplete solvent restoration and/or the carryover of residual extracted compounds that progressively altered solvent quality (Muñoz-Seijas et al., 2025).

The ATR–FTIR spectra of the recovered NADES after each reuse cycle (DES1–DES3) remained highly consistent, indicating that the choline chloride: glycerol hydrogen-bonded structure was largely preserved during repeated extraction–precipitation and solvent-recovery steps (Fig. 4b). All recycled samples exhibited the characteristic broad O—H stretching band (∼3200–3500 cm^−1^), C—H stretching bands (∼2930–2850 cm^−1^), and conserved fingerprint features (∼1150–950 cm^−1^), with no new absorption bands or pronounced peak shifts across cycles. Similar observations were reported by (Sui et al., 2023), who found that regenerated choline chloride-based DES components retained their main ATR–FTIR features after membrane-based recovery. Minor spectral differences in the present study were limited to small intensity/baseline variations, likely reflecting subtle changes in solvent composition or hydrogen-bonding environment rather than structural degradation.

Overall, the ChCl:Gly NADES retained a substantial proportion of its extraction capacity after reuse, maintaining > 85% performance after three cycles while preserving its FTIR signature. From a process sustainability standpoint, the combination of high solvent recovery (∼90 %) and acceptable extraction efficiency retention supports the feasibility of short-term closed-loop operation. For longer-term or larger-scale implementation, the viscosity build-up highlighted here indicates that periodic control of solvent composition may be required to stabilize mass transfer and maintain extraction performance.

## 4 Conclusion

This study shows that the precipitation route largely determines how effectively soy proteins can be recovered from choline chloride: glycerol NADES extracts. Single-step approaches revealed clear selectivity limits. Isoelectric precipitation maximized recovery, while ethanol-containing antisolvents improved phase separation compared with water, which was ineffective at low dilution. The combined strategy delivered the most balanced performance, achieving ∼77 % recovery and ∼89 % purity, with the highest precipitation yield among the tested conditions. FTIR showed no detectable changes in the main protein backbone bands, while SEM revealed broadly similar dried powder morphologies with some route-dependent differences. Importantly, the NADES could be recovered and reused for three cycles, achieving high mass recovery (∼90 %) and acceptable retention of extraction capacity (>85 %), with the main limitation being viscosity buildup that likely constrained mass transfer. These findings provide a practical basis for integrating protein recovery and solvent reuse in NADES-based plant protein processing, while highlighting the need for viscosity/composition control in extended recycling. Although the recovered proteins retained their main structural characteristics, further investigation of functional properties, such as solubility, emulsifying activity, and foaming capacity, would provide valuable insights into their potential applicability in food and industrial formulations.

Future studies may focus on strategies to stabilize solvent viscosity during prolonged recycling and on evaluating the scalability of the precipitation–recovery process in continuous or pilot-scale extraction systems.

## CRediT authorship contribution statement

**Gulay Ozkan:** Writing – review & editing, Writing – original draft, Visualization, Methodology, Investigation, Formal analysis, Data curation. **Gulsah Karabulut:** Writing – review & editing, Writing – original draft, Visualization, Methodology, Investigation, Formal analysis, Data curation.

## Declaration of competing interest

The authors declare that they have no known competing financial interests or personal relationships that could have appeared to influence the work reported in this paper.

## Data Availability

Data will be made available on request.
